# Supplementary material for: Diagnostic performance of capillary and venous blood samples in the detection of Loa loa and Mansonella perstans microfilaraemia using light microscopy
Source: PLoS Negl Trop Dis. 2021 Aug 16;15(8):e0009623. doi: 10.1371/journal.pntd.0009623 (PMC8389422; doi:10.1371/journal.pntd.0009623)
Supplement: Attachment — Submitted filename: VenCapWorm_AuthorResponses.docx [file pntd.0009623.s002.docx]

Diagnostic performance of capillary and venous blood samples in the detection of Loa loa and Mansonella perstans microfilaraemia using light microscopy

Dear editor

We are grateful for your message and the reviewers’ comments which we followed in great detail.

Please find below a point-by-point response to each reviewer comment.

We attached a revised clean version of the manuscript (no track changes), as well as, a version of the manuscript in which changes have been highlighted in color:

- pink + crossed: Passage was deleted

- yellow: Passage was modified or added

Furthermore, we would like to confirm that Michael Ramharter is the correspondence author of this article. This does not constitute a change between the first and the revised version of the manuscript, as Michael Ramharter was named correspondence author already on the title page of the first submission.

Once more we would like to thank the reviewers for their fruitful feedback which we believe improved the quality of the manuscript.

Thank you and best wishes,

Michael Ramharter

Johannes Mischlinger

**Responses to Editor Comments**

Editor:

1) We note that the following authors have not yet confirmed their authorship on this manuscript: Rella Zoleko Manego

Nina Hackbarth

Pierre Blaise Matsiegui

Ayôla Akim Adegnika

Selidji Todagbe Agnandji

At this point this request is not urgent; we have re-sent their co-author confirmation email containing an auto-confirmation link for them to click, and we will reach out to them at a later stage if we don't hear from them. We wanted to mention this in case you are aware of them experiencing technical issues with confirming authorship via the link. If so, please do ask them to contact us directly via email and we'd be happy to assist.

Author response: We thank the editor of pointing this out. We are not aware that any of these colleagues experience technical difficulties. However, we have contacted these authors to check their e-mails so that they can respond to the electronic authorship confirmation.

Editor:

2) Please ensure that the CRediT author contributions listed for every co-author are completed accurately and in full.

At this stage "all Authors" require contributions. Please ensure that the full contributions of each author are acknowledged in the ""Add/Edit/Remove Authors"" section of our submission form.

Author response and changes: We modified the author contributions section in the manuscript to comply with the CRediT taxonomy. Furthermore, we acknowledged the individual authors’ roles also in the submission form.

Editor:

3) Please provide an Author Summary. This should appear in your manuscript between the Abstract (if applicable) and the Introduction, and should be 150–200 words long. The aim should be to make your findings accessible to a wide audience that includes both scientists and non-scientists. Sample summaries can be found on our website under Submission Guidelines.

Author response: We added an author summary as requested.

Changes:

**Author summary:**

Microfilaraemia of Loa loa and Mansonella perstans was investigated by light microscopy in paired thick smears of capillary and venous blood; each sample was prepared using a standardised quantity of 10 microliters of blood and analysed by a minimum of two independent microscopists. Microfilaraemia was on average +34.5% (95% CI: +11.0 to +63.0) higher in capillary than in venous blood samples for L. loa and +24.8% (95% CI: +0.0 to +60.5) for M. perstans. This might have implications for treatment algorithms of onchocerciasis and loiasis, in which exact quantification of L. loa microfilaraemia is of importance. Furthermore, the odds for detection of M. perstans microfilariae was 65% higher in capillary than in venous blood which may pre-dispose capillary blood for detection of M. perstans infection in large epidemiological studies when sampling of large blood quantities is not feasible. No solid evidence for a higher odds of L. loa microfilariae detection in capillary blood was revealed, which might be explained by generally high levels of L. loa microfilaraemia in capillary and venous blood.

Editor:

4) We noticed that you used "unpublished data" in the manuscript. We do not allow these references, as the PLOS data access policy requires that all data be either published with the manuscript or made available in a publicly accessible database. Please either remove these references, or amend the supplementary material to include the referenced data.

Author response: We thank the editor for pointing this out. We removed the reference.

Changes: The sentence “The magnitude of this effect stems from incidental findings of earlier studies from the target region (unpublished data).” was deleted.

Editor:

5) Thank you for including your ethics statement: "Participants or their legal representatives gave informed consent before any study related procedures were performed". Please state whether written/verbal consent was obtained from the participants.

Author response: We thank the editor for pointing this out. Written consent was obtained from all participants. We included this in the ethics section.

Changes:

**Methods:**

This study was approved by the ethics committee of CERMEL under the number CEI-013/2018. Participants or their legal representatives gave written informed consent before any study related procedures were performed.

**Responses to Reviewer Comments**

<b>Methods</b></br></br>
-Are the objectives of the study clearly articulated with a clear testable hypothesis stated?</br>
-Is the study design appropriate to address the stated objectives?</br>
-Is the population clearly described and appropriate for the hypothesis being tested?</br>
-Is the sample size sufficient to ensure adequate power to address the hypothesis being tested?</br>
-Were correct statistical analysis used to support conclusions?</br>
-Are there concerns about ethical or regulatory requirements being met?</br></br>

Reviewer #1: The objective of the study conducted by Mischlinger et al. was to compare the number of Loa loa and Mansonella perstans microfilariae observed in 10 µL of venous blood and 10 µL of capillary blood. The authors found that the microfilarial densities (MfD) were significantly higher in capillary blood and state that this finding is important because it “might have implications for treatment algorithms of onchocerciasis and loiasis, in which exact quantification of L. loa microfilaraemia is of importance”.

Reviewer #1:
The authors say their study is the first to be conducted to compare Loa and M. perstans MfDs in venous and capillary blood. This is not correct. Kershaw (Ann Trop Med Parasitol 1950) studied this on two subjects (and found that the L. loa and M. perstans MfDs are higher in capillary blood). Similarly, Noireau et al. (J Trop Med Parasitol 1990) compared L. loa and M. perstans MfDs in venous and capillary blood collected from 201 subjects. In addition, the fact that the MfD is higher in capillary blood than in venous blood is also very well known for Wuchereria bancrofti, the cause of lymphatic filariasis, whose microfilariae are similar in size to those of L. loa. It is very strange that the authors did not review the studies comparing different methods used to quantify W. bancrofti MfD from venous and capillary blood. One of the first articles is that of Yorke & Blacklock, Ann Trop Med Parasitol 1917. Over 15 other articles present data on this subject. This review should
have been included in the introduction or in the discussion section of the paper.
Author response:

We thank the reviewer for this comment with which we agree. We incorporated these references (including others) in the manuscript and deleted all sentences that could be interpreted as claim of primacy. Changes were made in the introduction and discussion sections.

Changes:

**Introduction:**

About half of the introduction section was rewritten:

“In the past century, several studies investigated the density of filarial blood parasites in samples of capillary (CAP) or venous (VEN) blood over a 24-hour cycle to allow studying of potential circadian periodicities. Most of this research was done for lymphatic filariasis, however, to a lesser extent also for infections with L. loa and certain species of Mansonella [9–18]. Two studies investigated the microfilarial density and microfilaraemia prevalence, respectively of L. Loa and M. perstans infections in paired samples of CAP and VEN blood [17,18]. Such information is not merely of academic interest, as higher parasite densities in one blood type can facilitate higher chances of detection by diagnostic tools, as was recently demonstrated for malaria [19]. However, in spite of good methodological quality the findings of the first study are based on only two (n=2) infected individuals [17] and the second study recruited 39 and 97 individuals infected with L. loa and M. perstans, respectively [18]. Therefore, in order to add more evidence to this neglected field of research we conducted a large diagnostic study investigating the diagnostic performance characteristics of CAP and VEN blood to detect L. Loa and M. perstans in infected persons. This study quantified the microfilaraemia of L. loa and M. perstans in samples of CAP and VEN blood and compared the odds of microfilaria detection in both blood types.”

**Discussion:**

A paragraph was added:

Previous studies show concordant evidence in favour of higher L. loa and M. perstans microfilaraemia in CAP than in VEN blood samples when equal blood volumes were evaluated. In 1950, Kershaw assessed CAP and VEN blood samples of each 50 microliters blood taken from two participants and demonstrated a higher microfilarial blood density in CAP blood than in VEN blood [17]. Later, in 1990 Noireau and Apembet assessed the diagnostic sensitivity of 40 microliters of CAP blood samples in 39 and 97 individuals infected with L. loa and M. perstans, respectively whose infection status was ascertained by light microscopical assessment of 5ml VEN blood lysed with 2% saponin [18]. They indicated that 40 microliters of CAP blood had a sensitivity of 67% (26/39) and 62% (60/97) to detect L. loa and M. perstans, respectively. However, a direct comparison of diagnostic properties of CAP and VEN blood samples was not possible, as preparation methods and blood quantities were different prior to diagnostic assessment.

Reviewer #1:
The authors collected 10 µL of capillary blood and 10 µL of venous blood (without fully explaining how they proceeded for the venous blood). As they write themselves, this quantity is very low but they explain that it "was restricted by the small extractable quantities of CAP blood". This argument does not hold since the volume of blood routinely (and easily) taken by finger prick during filariasis surveys is 50 µL. It would have been more interesting to compare the MfDs in two 50 µL samples, which would have detected more subjects with low microfilaremia.
Author response:

We agree with the reviewer and also other reviewers for commenting that this passage may lead readers to think that 10 microliters are the maximally extractable blood quantity of CAP blood. This was not intentional and we thank the reviewer for pointing this out. In this study light microscopy was taken as diagnostic method. Although a gold-standard diagnostic tool in parasitology it is known that microscopy can yield subjective results. Therefore, to correct for this so-called inter-reader variability up to three microscopists analysed thick smear samples and arithmetic means of samples were taken as the definitive result. 10 microliters were chosen within this context, as this amount is what is approximately used for malaria diagnostics in routine health care settings. This was considered important given the objectives of the study to quantify and compare microfilaraemia in CAP and VEN blood when used in routine health care settings. Furthermore, a quantity of 10 microliters seems to allow to extrapolate study results to modern technology for rapid assessment of microfilariae (e.g. LoaScope); for example, the LoaScope technology is based on CAP blood collection using disposable rectangular capillaries made from plastic. Such rectangular or cylindrical plastic capillaries are common also in intensive care medicine and rarely hold more than 10 microliters of CAP blood. However, in spite of all arguments that might favour the assessment only 10 microliters we do declare this as a limitation as it increases the diagnostic limit of detection.

The discussion section was modified so that it does not seem, that CAP blood collection is restricted to a maximum of 10 microliters.

References:

D’Ambrosio M V, Bakalar M, Bennuru S, Reber C, Skandarajah A, Nilsson L, et al. Point-of-care quantification of blood-borne filarial parasites with a mobile phone microscope. Sci Transl Med. 2015 May;7(286):286re4.

Kamgno J, Pion SD, Chesnais CB, Bakalar MH, D’Ambrosio M V, Mackenzie CD, et al. A Test-and-Not-Treat Strategy for Onchocerciasis in Loa loa-Endemic Areas. N Engl J Med. 2017 Nov;377(21):2044–52.

Changes:

**Discussion:**

This study only investigated 10 microliters of blood, a blood quantity which is common particularly for diagnosis of malaria in clinical and research settings. This constitutes a limitation, as it is possible to extract higher quantities of CAP blood during a given CAP blood sampling session (e.g. 50 microliters). Furthermore, in individual case management when infection by a filarial blood parasite is suspected it is recommended to withdraw venous blood of larger quantities (i.e. several milliliters) for microfilariae detection at different days [7,18]

Reviewer #1:
The statistical methods used to analyse the data are relatively complex. The authors write rightly that the logarithm of 0 does not exist but it is common in the field of filariasis to add 1 to the MfD to be able to calculate geometric means taking into account subjects with negative results (Williams’ mean).
Author response: We thank the reviewer for this comment and agree that it is important to solve this mathematical problem prior to statistical analysis. However, we think that the manuscript already is in agreement with the reviewer comment. Although we first mention that ”Log-transforming of 0-values is mathematically impossible ...” the next sentence introduces the strategy that was used to circumvent this problem i.e. “... a common strategy from the field of microbiology was applied ...”. This strategy advocates allocating a value of half the limit of detection (LOD) and ensures that geometric means can computed that allow for inclusion of subjects with negative results, similar to the Williams’ mean. On the other hand, Williams’ mean is at comparatively higher risk of producing artificial outliers due to log(x + 1) instead of log(x + [LOD/2]).

Changes:

No changes were made in the manuscript.

Reviewer #2: The objectives were clearly stated and the study design was appropriate, the study population was also clearly described and appropriate though the adequate sample size was not achieved to ensure adequate power (acknowledged by the researchers)
Ethical statement, should state the ethics committee approval number.
There are problems with clarity especially in the methods and results section for non-specialists. The authors need an English specialist to review that section and correct some grammatical presentations.

Author response: We thank reviewer for his/her comments. We reported the ethics committee approval number and revised the manuscript for enhanced clarity. A colleague of ours who is a UK English-native speaker working in London as infectious disease specialist proof-read the final version of the manuscript.

Changes:

**Abstract – Introduction:**

- It was recently established that the odds for detecting Plasmodium spp. is higher in capillary (CAP) blood than in venous (VEN) blood.

**Methods section:**

- Microscopists were blinded to each other's results.

- Differences created from two log-transformed continuous variables constitute a ratio after anti-log operations; such ratios allow to visualise the excess microfilaraemia in CAP blood relative to the corresponding VEN microfilaraemia expressed in percent

- Contingency tables were created to visualise the distribution of individual prevalence of CAP and VEN microfilaraemia. Odds ratios for paired data were computed to assess the odds of microfilaraemia detection in CAP versus VEN blood samples.

- Furthermore, diagnostic sensitivity of CAP and VEN samples was computed by using a light microscopy gold standard.

- This study was approved by the ethics committee of CERMEL under the number CEI-013/2018.

**Results section:**

- About a quarter (169/710) was below 10 years of age, 17% (120/710) in age group 10-19 years and approximately 10% in every subsequent 10-year age band, ending with 13% (93/710) in the age group of 70 years and older.

- For individuals with L. loa infection median (IQR) CAP microfilaraemia was 3,650 (275-11,100) per milliliter blood and VEN microfilaraemia was 2,775 (200-8,875) with strong evidence for a true CAP-VEN difference in the target population (p<0.0001) (TABLE 2).

**Discussion section:**

- However, it is of mention that to the best of our knowledge this is the largest study to date on this topic.

- Applying the exact same diagnostic methods and investigating equal volumes of blood we noted that L. loa microfilaraemia was about 33% higher in CAP blood than in VEN blood (p=0.0027) and about 25% higher for M. perstans infection (p=0.08).

**Conclusion section:**

- This might be explained by the overall high levels of L. loa microfilaraemia in both CAP and VEN blood which were generally above the microscopic limit of detection in both blood sources; also, it is possible that the study was underpowered to detect such an effect.

Reviewer #3: (No Response)

--------------------

<b>Results</b></br></br>
-Does the analysis presented match the analysis plan?</br>
-Are the results clearly and completely presented?</br>
-Are the figures (Tables, Images) of sufficient quality for clarity?</br></br>

Reviewer #1: The analysis presented match the analysis plan and the results are clearly and completely presented.
Author response: We thank the reviewer for this comment.

Changes: No changes were made.

Reviewer #2: Yes the analysis presented match the analysis plan but the prose of the results needs to be made clearer. On the other hand, the tables are clearly presented.
Author response:

We thank the reviewer for this comment and we tried our best to make the prose in the results section clearer. The applied changes are summarised below. However, it is difficult to infer from the reviewer comment what precisely should be modified. We acknowledge to deviate from the common approach of merely reporting results as “significant” or “non-significant”, but rather interpreting p-values as measuring the statistical strength of evidence against the null-hypothesis. This strength of evidence against the null-hypothesis can also be interpreted as the probability that a given study finding is only due to chance; a p-value of 0.5 would indicate a probability of 50% that a given finding would be due to chance and that this finding might actually be very different (or not exist at all) in the larger target population. This conceptual framework constitutes the rationale for the prose in the results section. However, this was largely done on purpose in accordance with current curricula of academic courses on epidemiology (4 authors hold an epidemiology degree by the London School of Hygiene and Tropical Medicine) which strongly encourage researchers to adhere to the afore-mentioned approach. Furthermore, the last decade has seen publications in high-ranking journals many of which also support a more accurate statistical reporting that incorporates strengths and limitations of the given statistical methodology (references are cited below). Many of such publications highlight the need of more differential reporting related to results with a p-value in the range between 0.01 and 0.1 (e.g. Sterne and Smith 2001); this is the case for some findings in our study. We tried to follow these current suggestions of more detailed reporting of statistical aspects related to findings of a study.

We agree that some passages (e.g. paragraph 4: “Contingency tables demonstrate that the number of observations was higher in the discordant pair of ‘CAP+ & VEN-‘ ...”) are somewhat technical and may be perceived as complex by some readers. However, in our opinion this owes to the applied statistical methodology; we computed ‘odds ratios for paired data’ and MCNemar tests which are derived from the discordant pairs in a contingency table. This methodology may be less known in the overall research community, yet it constitutes the statistical gold standard methodology for comparison of diagnostic samples when it is not clear beforehand what diagnostic sample has better diagnostic properties. Given the prominence of these afore-mentioned discordant pairs in the analysis it is common to present the absolute numbers of the respective discordant pairs in the main text. Changing this would in our opinion lead to a counter-intuitive result which is that the results section would become less well readable. Therefore, as it was not specified what precise changes shall be implemented we hope that it is acceptable to the reviewer and the editor that the overall presentation of findings stays similar in this revised version of the manuscript. The structure of the results section follows a logical approach i.e. A) Baseline characteristics B) Description of absolute differences in CAP-VEN microfilaraemia; first medians (i.e. non-parametric analyses) then linear models (i.e. parametric analyses) C) Description of discordant pairs including odds ratios for paired data D) Sensitivity analyses

References:

Sterne JA, Davey Smith G. Sifting the evidence-what’s wrong with significance tests? BMJ. 2001 Jan;322(7280):226–31.

Amrhein V, Greenland S, McShane B. Scientists rise up against statistical significance. Nature. 2019 Mar;567(7748):305–7.

Ioannidis JPA. Retiring statistical significance would give bias a free pass. Vol. 567, Nature. England; 2019. p. 461.

Changes:

**Results:**

- About a quarter (169/710) was below 10 years of age, 17% (120/710) in age group 10-19 years and approximately 10% in every subsequent 10-year age band, ending with 13% (93/710) in the age group of 70 years and older.

- For individuals with L. loa infection median (IQR) CAP microfilaraemia was 3,650 (275-11,100) per milliliter blood and VEN microfilaraemia was 2,775 (200-8,875) with strong evidence for a true CAP-VEN difference in the target population (p<0.0001) (TABLE 2).

- Among individuals with M. perstans infection a ranked comparison of paired CAP and VEN microfilaraemia indicated similar results with the median (IQR) being identical at 100 (0-200) (p=0.44).

- Contingency tables demonstrate that the number of observations was higher in the discordant pair of ‘CAP+ & VEN-‘ than in the pair of ‘CAP- & VEN+’ both for infections with L. loa and M. perstans (TABLE 4). This indicates that detection of microfilaraemia was more common in CAP than in VEN blood samples. However, while hypothesis tests demonstrate no evidence in support of this finding for L. loa infection (‘CAP+ & VEN-‘: 21 and ‘CAP- & VEN+’: 17; p=0.52) they provide such evidence for M. perstans infection (‘CAP+ & VEN-‘: 43 versus ‘CAP- & VEN+’: 26; p=0.041).

Reviewer #3: (No Response)

--------------------

<b>Conclusions</b></br></br>
-Are the conclusions supported by the data presented?</br>
-Are the limitations of analysis clearly described?</br>
-Do the authors discuss how these data can be helpful to advance our understanding of the topic under study?</br>
-Is public health relevance addressed?</br></br>

Reviewer #1: By reading the literature on W. bancrofti mentioned above, the authors could have discussed the hypotheses proposed by the various authors who have worked on the subject to explain the phenomenon of higher MfD in capillary blood. In the manuscript, they just say that in malaria the differences “can be explained by host-parasite receptor interactions”. However, it is clear that the microfilariae are large organisms (unlike Plasmodium, to which the authors refer several times) and that the observed differences are probably at least partly due to the ratios between the size of the parasites and the diameter of blood vessels.
Author response:

We agree with the reviewer and incorporated existing hypotheses for higher CAP microfilarial densities in the discussion section.

Changes:

**Discussion:**

In 1917, Yorke and Blacklock discussed that microfilariae of Wuchereria bancrofti are more abundant in CAP than in VEN blood irrespective of varying microfilarial peak densities during a 24-hour cycle. This phenomenon was explained by mechanical obstruction of microfilariae during passage through the small cutaneous capillary blood vessels aiding in the piling up of larvae in the cutaneous vessels [9]. It is likely that this explanation also applies to microfilariae of L. loa and M. perstans which are similar in size (about 275x7μm and 210x4μm, respectively) [1,2].

Reviewer #1:
When the authors say that their findings may have implications for the treatment of onchocerciasis and loiasis because it is necessary to know the exact Loa MfD before giving treatment, they overestimate somewhat the interest of their results. Actually, the decisions are made according to the MfD measured by the reference method which is the quantification in capillary blood (which enables to detect at-risk subjects with high MfD). The fact that the density is lower in the venous blood does not really matter.
Author response:

We thank the reviewer for this critical comment and we attempted to identify and read all available evidence on this topic. By reading the main publications on LoaScope, which currently is considered the most promising tool for onchocerciasis treatment algorithms (references cited below), it is not at all obvious that exclusively CAP blood sampling is advocated. Certainly, we agree with the reviewer that CAP blood may already be used as the main blood type for rapid assessments, because its sampling is simpler than for VEN blood. However, any potential safety argument in favour of CAP blood usage in LoaScope was hitherto not at all robust, as the only study that investigated *L. loa* microfilaraemia before in CAP-VEN samples of equal volume was published by Kershaw in 1950; the results of this study are based on only two infected individuals. Therefore, in our opinion this current study constitutes an important piece of confirmatory science that corroborates earlier preliminary evidence that *Loa loa* microfilaraemia is higher in CAP than in VEN blood. In spite of the fact that the target sample size was not achieved for *M. perstans* (n=120) (N.B.: It was achieved for *L. loa*) this study includes the to date highest number of participants with microscopically-confirmed *L. loa* (n=152) and *M. perstans* microfilaraemia (n=102) that investigated CAP and VEN microfilaraemia. Therefore, we believe that this current study gives important insights into safety considerations for current treatment algorithms, but also for potential, future treatment algorithms.

We modified the discussion section to indicate CAP blood might already be used for the majority of rapid blood assessments due to feasibility reasons, however, that this current study also adds a safety argument in favour of CAP blood sampling.

References:

D’Ambrosio M V, Bakalar M, Bennuru S, Reber C, Skandarajah A, Nilsson L, et al. Point-of-care quantification of blood-borne filarial parasites with a mobile phone microscope. Sci Transl Med. 2015 May;7(286):286re4.

Kamgno J, Pion SD, Chesnais CB, Bakalar MH, D’Ambrosio M V, Mackenzie CD, et al. A Test-and-Not-Treat Strategy for Onchocerciasis in Loa loa-Endemic Areas. N Engl J Med. 2017 Nov;377(21):2044–52.

Changes:

**Discussion:**

Therefore, in order to ensure safety of ivermectin mass drug administration CAP blood should be used for rapid quantification of L. loa. microfilariae with LoaScope. It is likely that CAP blood sampling may already constitute the main blood sampling method for rapid assessments, as it is an easy procedure even under field conditions. However, besides this feasibility aspect this current study adds an additional safety argument in favour of CAP blood sampling.

Reviewer #2: The conclusions are supported by the presented results, and the limitations of analysis clearly described. The authors have discussed adequately the implications of the data presented and its relevnce to public health.
Author response: We thank the reviewer for this comment.

Changes: No changes were made.

Reviewer #3: (No Response)

--------------------

<b>Editorial and Data Presentation Modifications?</b></br><br/>
Use  this section for editorial suggestions as well as relatively minor modifications of existing data that would enhance clarity. If the only modifications needed are minor and/or editorial, you may wish to recommend “Minor Revision” or “Accept”. 

Reviewer #1: Line 83, delete “(L.)” and “(M.)”
Author response and changes: “(L.)” and “(M.)” were deleted in line 83

Line 85, delete both “spp.”
Author response and changes: Both “spp.” were deleted in line 88

Line 97: cite Asio et al., Parasitology Research 2009
Author response and changes: Reference was cited

Line 103: in the list of references, reference 8 is incomplete

Author response and changes: We thank the reviewer for pointing out on this. Reference 8 was completed.
Line 140: check reference number
Author response and changes: We thank the reviewer for pointing out on this. These references date back to an earlier manuscript version. We corrected this.

Reviewer #2: The sample size determination should come before materials and parasitological analysis, not after statistical considerations.
Author response and changes: Following the reviewers comment we inserted sample size determination before materials and parasitological analysis.

Reviewer #3: (No Response)

--------------------

<b>Summary and General Comments</b></br></br>
Use  this section to provide overall comments, discuss strengths/weaknesses of the study, novelty, significance, general execution and scholarship. You may also include additional comments for the author, including concerns about dual publication, research ethics, or publication ethics. If requesting major revision, please articulate the new experiments that are needed.

Reviewer #1: This paper is of limited interest. The results confirm those presented in other papers on Loa loa, M. perstans, and W. bancrofti. They are not very original and will not have any implication on the strategies to combat onchocerciasis in loiasis-coendemic areas.
Author response: We appreciate the reviewer’s helpful comments, particularly those that pointed out to us that we needed to incorporate more evidence on previous research done on filarial parasitic diseases. However, after having incorporated earlier research the relevance of our study seems even higher, as earlier studies on this topic were often descriptive in nature and based on a very low number of participants. Therefore, in our opinion this study constitutes an important piece of confirmatory science for earlier preliminary evidence that *Loa loa* microfilaraemia is higher in CAP than in VEN blood. In spite of the fact that sample size was not achieved for M. perstans this study includes that to date highest number of participants with microscopically-confirmed M. perstans microfilaraemia that investigated CAP and VEN microfilaraemia.

Due to all above-mentioned we further believe that research findings are important also for current strategies and treatment algorithms for onchocerciasis (e.g. LoaScope). Even if it might already be commonplace to sample finger prick blood for feasibility and convenience reasons a solid evidence basis in favour of CAP blood sampling due to a statistically significant and clinically relevant higher microfilaraemia in CAP blood was established by this current study. However, in accordance with the evidence that we inserted in the manuscript we also rewrote this passage and believe that this led to an improved quality.

Changes:

**Discussion:**

- Previous studies show concordant evidence in favour of higher L. loa and M. perstans microfilaraemia in CAP than in VEN blood samples when equal blood volumes were evaluated. In 1950, Kershaw assessed CAP and VEN blood samples of each 50 microliters blood taken from two participants and demonstrated a higher microfilarial blood density in CAP blood than in VEN blood [17]. Later, in 1990 Noireau and Apembet assessed the diagnostic sensitivity of 40 microliters of CAP blood samples in 39 and 97 individuals infected with L. loa and M. perstans, respectively whose infection status was ascertained by light microscopical assessment of 5ml VEN blood lysed with 2% saponin [18]. They indicated that 40 microliters of CAP blood had a sensitivity of 67% (26/39) and 62% (60/97) to detect L. loa and M. perstans, respectively. However, a direct comparison of diagnostic properties of CAP and VEN blood samples was not possible, as preparation methods and blood quantities were different prior to diagnostic assessment. Applying the exact same diagnostic methods and investigating equal volumes of blood we noted that L. loa microfilaraemia was about 33% higher in CAP blood than in VEN blood (p=0.0027) and about 25% higher for M. perstans infection (p=0.08).

- In the past, the implementation of ivermectin-based mass drug administration for the control of onchocerciasis or lymphatic filariasis has been impaired because of afore-mentioned serious adverse events in persons with a high L. loa microfilaraemia (i.e. > 20,000 microfilariae/milliliter blood) [27]. To overcome this treatment gap, a so called ‘Test and not treat’ strategy was developed which is based on withholding mass drug administration from those people with high levels of L. loa microfilariae [28,29]. Such highly microfilaraemic persons are identified by means of rapid testing with a diagnostic tool named LoaScope, which analyses samples of peripheral blood [28,30]. Therefore, in order to ensure safety of ivermectin mass drug administration CAP blood should be used for rapid quantification of L. loa. microfilariae with LoaScope. It is likely that CAP blood sampling may already constitute the main blood sampling method for rapid assessments, as it is an easy procedure even under field conditions. However, besides this feasibility aspect this current study adds an additional safety argument in favour of CAP blood sampling.

Reviewer #2: The study adds some value to the body of knowledge already known in this area. Identifying that capillary blood samples yield higher levels of parasitaemia has been known for malaria, but not for microfilaria. That is what this study fills in.
The details of the methodology are sufficient to reproduce the study
There are problems with clarity especially in the methods and results section for non-specialists. The authors need an English specialist to review that section and correct some grammatical presentations. Major ones have been highlighted here. The sample size determination should come before materials and parasitological analysis, not after statistical considerations.

Author response and changes:

We thank the reviewer for this comment. The ‘sample size’ section was inserted before ‘materials and parasitological analysis’. Furthermore, as already indicated in an earlier response to a comment by reviewer #2, we revised the manuscript for enhanced clarity. A colleague of ours who is a UK English-native speaker working in London as infectious disease specialist proof-read the final version of the manuscript. Specific changes were already highlighted before.

Reviewer #2:
•	The academic achievements should be removed from the authors names

Author response and changes: Academic achievements were removed from the authors names

Reviewer #2:
•	Line 117, ‘two thick smears of blood, 10 microliters each were prepared per participants’
•	Line 118, ‘one from capillary and one from venous blood at the same time’

Author response and changes: Lines 117+118 were modified and now read as follows: “Thick smears of 10 microliters of blood were prepared and stained with 4% Giemsa for 60 minutes. For each participant two thick smears were prepared, one from CAP and one from VEN blood sampled at the same time.”

Reviewer #2:
•	Line 162, ‘120 participants each with L. loa infection and 120 with M. perstans infection are required’

Author response and changes: The sentence was modified as suggested: “Thus, in order to answer both primary objectives of the study 120 participants each with L. loa infection and 120 with M. perstans infection are required who are positive for the respective filarial infection in either CAP or VEN blood.”

Reviewer #2:
•	Ethical statement, should state the ethics committee approval number

Author response and changes: The ethics committee approval number was stated.

Reviewer #2:
•	Line 170, participants were

Author response and changes: The change was made as suggested.

Reviewer #2:
•	Line 172, subsequent 10-year age group

Author response and changes: The change was made as suggested. The sentence reads as follows: “About a quarter (169/710) was below 10 years of age, 17% (120/710) in age group 10-19 years and approximately 10% in every subsequent 10-year age band, ending with 13% (93/710) in the age group of 70 years and older.”

Reviewer #2:
•	Line 182 remove revealed

Author response and changes: The word “revealed” was removed from the sentence.

Reviewer #2:
•	Line 282 largest study to date

Author response and changes: This passage was deleted in the revised version of the manuscript.

Reviewer #2:
•	Information in line 303, is better put in the methods section

Author response and changes: This passage was modified in the revised version of the manuscript.

Reviewer #2:
•	Lines 327, 338, the references are incomplete
Author response and changes: References in lines 327 and 338 were completed.

Reviewer #3: The paper by Mischlinger et al. presents the results of comparing measurements of microfilariae of Loa loa and Mansonella perstans between venous and capillary blood collected from 201 patients.

The paper presents an incomplete bibliography as there are two papers that have already performed this experiment; and a large number of papers on Wuchereria bancrofti. The authors state that this is the first experiment, which is not the case. The authors should complete the bibliography accordingly. Instead, the comparison with malaria is throughout the paper, which is less coherent.

Author response:

We thank the reviewer for this comment with which we agree. We incorporated many more research papers on filarial diseases in this manuscript and believe that this has led to a much-improved version of the manuscript. Furthermore, the in-depth comparison to malaria was deleted from the discussion section.

Changes:

**Introduction:**

About half of the introduction section was rewritten:

“In the past century, several studies investigated the density of filarial blood parasites in samples of capillary (CAP) or venous (VEN) blood over a 24-hour cycle to allow studying of potential circadian periodicities. Most of this research was done for lymphatic filariasis, however, to a lesser extent also for infections with L. loa and certain species of Mansonella [9–18]. Two studies investigated the microfilarial density and microfilaraemia prevalence, respectively of L. Loa and M. perstans infections in paired samples of CAP and VEN blood [17,18]. Such information is not merely of academic interest, as higher parasite densities in one blood type can facilitate higher chances of detection by diagnostic tools, as was recently demonstrated for malaria [19]. However, in spite of good methodological quality the findings of the first study are based on only two (n=2) infected individuals [17] and the second study recruited 39 and 97 individuals infected with L. loa and M. perstans, respectively [18]. Therefore, in order to add more evidence to this neglected field of research we conducted a large diagnostic study investigating the diagnostic performance characteristics of CAP and VEN blood to detect L. Loa and M. perstans in infected persons. This study quantified the microfilaraemia of L. loa and M. perstans in samples of CAP and VEN blood and compared the odds of microfilaria detection in both blood types.”

**Discussion:**

A paragraph was added:

Previous studies show concordant evidence in favour of higher L. loa and M. perstans microfilaraemia in CAP than in VEN blood samples when equal blood volumes were evaluated. In 1950, Kershaw assessed CAP and VEN blood samples of each 50 microliters blood taken from two participants and demonstrated a higher microfilarial blood density in CAP blood than in VEN blood [17]. Later, in 1990 Noireau and Apembet assessed the diagnostic sensitivity of 40 microliters of CAP blood samples in 39 and 97 individuals infected with L. loa and M. perstans, respectively whose infection status was ascertained by light microscopical assessment of 5ml VEN blood lysed with 2% saponin [18]. They indicated that 40 microliters of CAP blood had a sensitivity of 67% (26/39) and 62% (60/97) to detect L. loa and M. perstans, respectively. However, a direct comparison of diagnostic properties of CAP and VEN blood samples was not possible, as preparation methods and blood quantities were different prior to diagnostic assessment.

Reviewer #3:
The authors should clarify in which framework this study was done. Indeed, it is obvious that this is an ancillary study and that it was not prepared for this purpose. In particular, this would explain why only 10 μL were collected from both venous and capillaries. It would be good to clarify this, and to put less emphasis on possible technical difficulties, as otherwise readers not used to this disease might think that it is difficult to perform capillary or venous sampling, which is not the case.
Author response:

We thank the reviewer for this comment and we wrote more about the context in which this study was performed. However, still this was an independent diagnostic study. Most of the authors have their main research focus on malaria, where it is common that two to three microscopists are involved in creating a diagnostic result. Usually, the arithmetic mean is taken based on results of two microscopists and only if the deviation between the two respective results is larger than 50% a third reader produces a last result and then an arithmetic mean is taken from the two samples that are closest to each other. This approach corrects for the subjectivity of light microscopy and reduces the problem of intra- and inter-microscopist variability. The decision of assessing only 10 microliters is mostly based on considerations of feasibility of the afore-mentioned two-to-three microscopist approach. Indeed, in other studies higher volumes of (VEN or CAP) blood were prepared and we also mentioned these 10 microliters as a limitation in our results section. However, we believe that for the comparison of CAP and VEN microfilaraemia it is primarily important to investigate equal sample quantities to enable a fair comparison. Furthermore, a quantity of 10 microliters seems to allow to extrapolate study results to modern technology for rapid assessment of microfilariae (e.g. LoaScope); for example, the LoaScope technology is based on CAP blood collection using disposable rectangular capillaries made from plastic. Such rectangular or cylindrical plastic capillaries are common also in intensive care medicine and rarely hold more than 10 microliters of CAP blood.

The discussion section was modified to better explain the decision to investigate only 10 microliters of blood. As in the first version of the manuscript this was declared a limitation of the study. Furthermore, it was clarified that it is possible to sample higher quantities of blood.

Changes:

**Methods:**

At the time of writing of the study protocol a similar diagnostic study in the field of malaria was ongoing at CERMEL [19]. Due to the sameness of the target population, as well as, the recruitment and analytical procedures, samples of the diagnostic malaria study were systematically re-analysed for L. loa and M. perstans and new participants were recruited in parallel.

**Discussion:**

This study only investigated 10 microliters of blood, a blood quantity which is common particularly for diagnosis of malaria in clinical and research settings. This constitutes a limitation, as it is possible to extract higher quantities of CAP blood during a given CAP blood sampling session (e.g. 50 microliters). Furthermore, in individual case management when infection by a filarial blood parasite is suspected it is recommended to withdraw venous blood of larger quantities (i.e. several milliliters) for microfilariae detection at different days [7,18]. Yet, it was the objective of this study to investigate CAP-VEN differences in microfilaraemia and a potentially resulting differential odds for microfilaraemia-detection. For that it was important to investigate equal sample volumes of CAP and VEN blood in order to enable a fair comparison. A quantity of 10 microliters was considered as feasible to be assessed in sufficient detail by several microscopists. The applied two-to-three microscopist approach is a methodological strength and ensures exact quantification of microfilaraemia so that measurement errors are considered minimal. Furthermore, it is a strength that all microscopists were blinded to each other’s results and received training on the diagnostic features of the two target microfilariae of L. loa and M. perstans.

Reviewer #3:
Last, it seems that the authors should more discuss about the raison about the observed difference.
Author response:

We agree with the reviewer and think him/her for this comment. We wrote more about the potential reason for this observed difference. In accordance with evidence identified in the literature (e.g. Yorke and Blacklock 1917) the to date most plausible explanation mechanism seems to be mechanical obstruction and piling up of the microfilariae in capillary blood.

References:

Yorke W, Blacklock B. Observations on the Periodicity of Microfilaria Nocturna. Ann Trop Med Parasitol [Internet]. 1917 Aug 23;11(2):127–48. Available from: https://doi.org/10.1080/00034983.1917.11684131

Changes:

**Discussion:**

In 1917, Yorke and Blacklock discussed that microfilariae of Wuchereria bancrofti are more abundant in CAP than in VEN blood irrespective of varying microfilarial peak densities during a 24-hour cycle. This phenomenon was explained by mechanical obstruction of microfilariae during passage through the small cutaneous capillary blood vessels aiding in the piling up of larvae in the cutaneous vessels [9]. It is likely that this explanation also applies to microfilariae of L. loa and M. perstans which are similar in size (about 275x7μm and 210x4μm, respectively) [1,2].

Reviewer #3:
Finally, the authors indicate that these results could have implications for control programmes, but this is difficult to relate. The samples used are always capillary, and the thresholds have been defined for this type of sampling, so, in my opinion, these results have no obvious implications.
In the end, although the analyses are well done, the paper may not be of sufficient interest for publication in Plos NTD.
Author response:

We thank the reviewer for this critical comment and we attempted to identify and read all available evidence on this topic. By reading the main publications on LoaScope, which currently is considered the most promising tool for onchocerciasis treatment algorithms (references cited below), it is not at all obvious that exclusively CAP blood sampling is advocated. Certainly, we agree with the reviewer that CAP blood may already be used as the main blood type for rapid assessments, because its sampling is simpler than for VEN blood. However, any potential safety argument in favour of CAP blood usage in LoaScope was hitherto not at all robust, as the only study that investigated *L. loa* microfilaraemia before in CAP-VEN samples of equal volume was published by Kershaw in 1950; the results of this study are based on only two infected individuals. Therefore, in our opinion this current study constitutes an important piece of confirmatory science that corroborates earlier preliminary evidence that *Loa loa* microfilaraemia is higher in CAP than in VEN blood. In spite of the fact that the target sample size was not achieved for *M. perstans* (n=120) (N.B.: It was achieved for *L. loa*) this study includes the to date highest number of participants with microscopically-confirmed *L. loa* (n=152) and *M. perstans* microfilaraemia (n=102) that investigated CAP and VEN microfilaraemia. Therefore, we believe that this current study gives important insights into safety considerations for current treatment algorithms, but also for potential, future treatment algorithms.

We modified the discussion section to indicate CAP blood might already be used for the majority of rapid blood assessments due to feasibility reasons, however, that this current study also adds a safety argument in favour of CAP blood sampling.

References:

D’Ambrosio M V, Bakalar M, Bennuru S, Reber C, Skandarajah A, Nilsson L, et al. Point-of-care quantification of blood-borne filarial parasites with a mobile phone microscope. Sci Transl Med. 2015 May;7(286):286re4.

Kamgno J, Pion SD, Chesnais CB, Bakalar MH, D’Ambrosio M V, Mackenzie CD, et al. A Test-and-Not-Treat Strategy for Onchocerciasis in Loa loa-Endemic Areas. N Engl J Med. 2017 Nov;377(21):2044–52.

Changes:

**Discussion:**

In the past, the implementation of ivermectin-based mass drug administration for the control of onchocerciasis or lymphatic filariasis has been impaired because of afore-mentioned serious adverse events in persons with a high L. loa microfilaraemia (i.e. > 20,000 microfilariae/milliliter blood) [27]. To overcome this treatment gap, a so called ‘Test and not treat’ strategy was developed which is based on withholding mass drug administration from those people with high levels of L. loa microfilariae [28,29]. Such highly microfilaraemic persons are identified by means of rapid testing with a diagnostic tool named LoaScope, which analyses samples of peripheral blood [28,30]. Therefore, in order to ensure safety of ivermectin mass drug administration CAP blood should be used for rapid quantification of L. loa. microfilariae with LoaScope. It is likely that CAP blood sampling may already constitute the main blood sampling method for rapid assessments, as it is an easy procedure even under field conditions. However, besides this feasibility aspect this current study adds an additional safety argument in favour of CAP blood sampling.
